# Effect of Scaling Task Constraints on the Learning Processes of Under-11 Badminton Players during Match-Play

**DOI:** 10.3390/children7100164

**Published:** 2020-10-04

**Authors:** Enrique Ortega-Toro, Juan Carlos Blanca-Torres, José María Giménez-Egido, Gema Torres-Luque

**Affiliations:** 1 Department of Physical Activity and Sport, Faculty of Sport Science, Regional Campus of International Excellence “Campus Mare Nostrum”, University of Murcia, 30107 Murcia, Spain; eortega@um.es (E.O.-T.); josemaria.gimenez@um.es (J.M.G.-E.); 2 Sports Performance Analysis Association, 30107 Murcia, Spain; 3 Department of Didactics of Musical, Plastic and Body Expression, Faculty of Humanities and Education Sciences, Campus Las Lagunillas, University of Jaén, 23071 Jaén, Spain; jcblancatorres@gmail.com (J.C.B.-T.); gtluque@ujaen.es (G.T.-L.)

**Keywords:** performance analysis, young players, equipment scaling, small-sided games, singles badminton

## Abstract

Scaling equipment and the playing space according to junior badminton players’ characteristics and needs is a key aspect to design optimal learning environments. The purpose of the study is to analyze the incidence of reducing the court size (from 13.40 m × 5.18 m to 11.88 m × 5.18 m) and net height (from 1.55 m to 1.30 m) for under-11 badminton players on the following technical and tactical variables: (a) service area; (b) stroke effectiveness; (c) kinds of technical strokes; (d) players’ hitting area; (e) shuttle landing area; (f) shuttle flight; and (g) rally length. Twenty-eight badminton players (mean age of players: 9.81 ± 0.93) were selected and played a badminton competition (B) with the current federative rules and a mini-badminton competition (MB) with the altered net height and court dimensions. The results showed that a lower net height and a shorter court would increase the frequency and variability of strokes and play patterns, introducing quantifiable changes considered beneficial for children in their first stages, both in training and competition.

## 1. Introduction

The creation of optimal learning environments that will allow players to improve their performance and motor skills acquisition at early stages is a key factor for a proper personal and athletic development [1,2]. Therefore, coaches and sport organizations should design child sports based on robust theoretical frameworks that understand the complexity of learning functional skills [2,3]. “Nonlinear pedagogy” (NLP), based on concepts from an ecological dynamics perspective, understand this complexity, due to the learning process rarely following a linear behavior progression [4], because each player uses different problem-solving strategies [1]. Moreover, the development and acquisition of functional motor skills respond to a process of self-organization to form stable patterns when the performer and environment interact [2,5,6,7].

According to Newell’s [6] constraints model, NLP elaborate five key principles for designing adequate environments to facilitate learning [2,4,5]: “representative learning design”; “developing relevant information-movement couplings”; “manipulation of constraints” (task, performer and environment constraints); “reducing conscious control of movement”; and “providing functional variability”. Thus, a meaningful learning design must occur in a real-sport context, which affords the optimal learning opportunities and manipulates the key constraints, influencing the performer’s intention to explore functional movements in problem-solving, while also focusing their attention on the effect of the action [8]. To do this, several studies pointed out the “manipulation of constraints” principle, and especially the task constraints, which is the most powerful principle for designing adequate learning environments, given their significance in learning [2,5,6]. Modifying the task constraints, such as the play rules, rules of the league, sport equipment, playing space, etc., could lead to the acquisition of certain functional movement patterns and decision-making, promoting variability and creativity [4,5,6,7]. Hence, NLP offers a pedagogical guideline to design optimal competitions, which improves the learning process [6,9,10].

The concept “scaling junior sport”, based on the aforementioned concepts, seeks to implement junior sport according to players’ characteristics and needs, and not just adapt the adult game to the young players, through the manipulation of the task constraints (ball size, court size, etc.) [11,12,13]. In this sense, some studies have been carried out in recent years in collective sports, such as soccer [13,14,15,16], basketball [17], handball [18], volleyball [19], field hockey [20], cricket [21] or rugby [22]. Whereas in racquet sports, most of the research was performed in tennis, to create or redesign the best environment and competition possible for junior tennis players [12]. Most of the studies have assessed the effect of reducing the net height [12,23,24,25,26,27], court size [12,23,24,26,27], playing with small racquets [28] or a low-compression ball [12,28,29] on child players during match-play. Results of these studies showed that the scaling of equipment and playing area improve the players’ match performance, engagement, enjoyment and the development of desirable movement patterns related to motor variability. However, in badminton, the research has focused to analyze the following aspects: technical and tactical [30], physiological [31,32] or performance analysis [33,34,35,36] in elite and junior elite players, and not so much in scaling the equipment and play space for junior players. Although, some associations and federations, such as the Spanish Badminton Federation [37], in a similar way to the tennis 10’s program developed by the International Tennis Federation, do structure badminton competitions for junior players, to manipulate key task constraints. In particular, a badminton junior program called “minibadminton” (MB) was developed, which is divided into three stages: under-9 (under 9 years old), under-11 (under 11 years old) and under-13 (under 13 years old) [37]. The MB program is characterized by significantly reducing the net height and court dimension at the first stage of MB (under-9) and increasing it progressively until the adult sports version.

However, to our knowledge, there is not much information about the effect of playing in scaled badminton competitions (MB) or under the same conditions as adult badminton (B). Furthermore, only two studies in a physical education context and one in a formal badminton competition have provided information on player learning when playing with scaled equipment and play spaces. The study developed by Nathan et al. [38] compared the NLP and Linear Pedagogy (LP) models during a badminton competition in students of approximately 13 years. The results showed that NLP improved the students’ game performance in terms of their tactical decision-making, recovery movement to base and skills execution regarding drop shots and smashes, as compared to LP. The second study investigated the effects of practice under Teaching Games for Understanding (TGfU), NLP and LP, indicating that students are more engaged during NPL or TGfU (age ~13 years old) than LP lessons [39]. Finally, the study conducted by Nor Azmi et al. [40] assessed the effect of playing in four different conditions, manipulating the racquet (40.0 cm to 35.0 cm), net (1.5 m to 1.2 m) and court size (13.40 m × 6.1 m). Four groups (*n* = 40, under-9 players) played in the following conditions: standard racquet, net and court size (SRSC); standard racquet, modified court and net (SRMC); modified racquet, standard court and net (MRSC); and modified racquet court and net (MRMC). This study has found that children’s hitting opportunities (number of strokes) and stroke effectiveness (strokes into designated areas) were higher using MRMC than in other conditions. These studies showed the overall results, so it is difficult to reach solid conclusions.

The key research question that will be tested here is whether MB, manipulating court size and net height, is likely to generate a greater amount and variability of technical and tactical behaviors than B. Therefore, the main purpose of the present study is to investigate the incidence of MB by reducing the court dimensions (from 13.40 m × 5.18 m to 11.88 m × 5.18 m) and net height (from 1.55 m to 1.30 m), also observing the differences with the current under-11 badminton players’ competition (B) in the following technical and tactical variables: (a) service area; (b) stroke effectiveness; (c) kinds of technical strokes; (d) players’ hitting area; (e) shuttle landing area; (f) shuttle flight; and (g) rally length. Hence, it could conceivably be hypothesized that MB will improve the use of different kinds of strokes (especially the “special strokes” classified into the observational instrument) [41], the stroke effectiveness, and will decrease the rally length, affording an offensive style of playing.

## 2. Materials and Methods

### 2.1. Design

The present research was a quasi-experimental and cross-sectional design [42]. To analyze the technical and tactical variables of the B and MB matches, an observational, nomothetic, multidimensional and continuous intra-sessional registration method was used [43]. This study respected the ethical principles established by the United Nations Educational, Scientific and Cultural Organization (UNESCO) Declaration on Bioethics and Human Rights and was approved for being developed by the Ethics Committee of the Local Institution (JUN.18/10). Taking into consideration the Declaration of Helsinki, all players, who participated voluntarily, had to submit an informed consent that was signed by their parents or legal guardians for the development of the study.

### 2.2. Participants

Twenty-eight badminton players were involved in the research. The sample selection was carried out through an intentional sampling method according to the criteria of accessibility and proximity (the specificity in the study design marked the non-randomized sample) [44]. With the purpose of controlling the internal validity of the sample, all players had to present similar characteristics (gender of players = 16 males and 12 females; age of players = 9.81 ± 0.93; and dominant hand = 27 right-handed and 1 left-handed). The study sample was the total number of strokes (*n* = 8888) made by the players between the B and MB matches.

**Figure 1:**
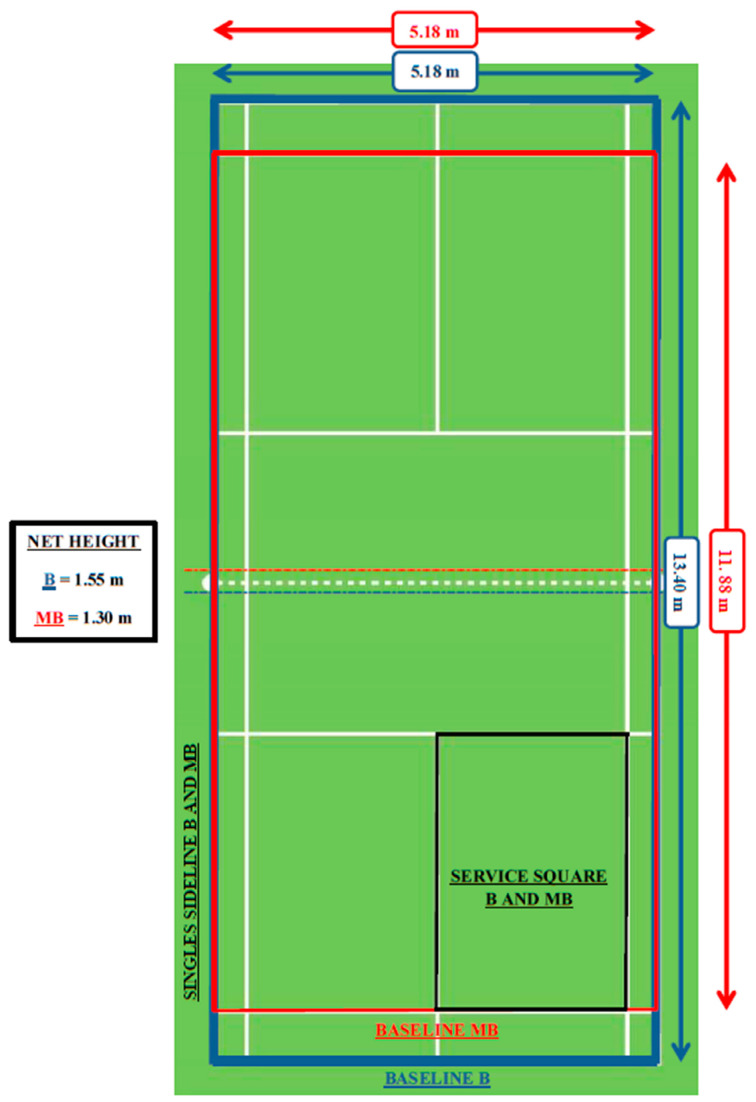
Illustration of net height and court dimensions in both competitions (Badminton formal format “B” = blue color and Mini-Badminton “MB” = red color).

To maintain stability between the B and MB, both competitions met the following common features: the distribution in the groups was carried out randomly because all the participants were elite kid badminton players at a similar level, so that each player was randomly assigned to a group of four players; the same number of total matches (42 matches) was played in each competition (badminton was played to the best of a 21-point set with a difference of 2 points up to a limit of 30 points and “minibadminton” was played to the best of a 15-point set with a difference of 2 points up to a limit of 21 points); both competitions were played with the round-robin system: each player played against the rest of the players in the group; that is to say, all players played the same number of matches. The matches were played in the same order and schedule; the average match duration was 14.30 ± 4.38 in MB and 24.97 ± 6.76 in B. The rest time between matches was at least the duration of a match plus 10 extra minutes (average rest between matches = 24.04 ± 4.53 in MB and 33.22 ± 6.73 in B) to avoid fatigue. The differences between B and MB were in the rules and equipment (score system, net height and court dimensions), which were defined by the Spanish Badminton Federation (FESBA) and the redesigning of a new format of the competition (Figure 1).

### 2.3. Instruments

The observational instrument for the Technical and Tactical Actions in Singles Badminton [41] was used according to the objective study in [45].

The observational instrument was implemented to analyze the players’ strokes across three key criteria: context, result and game. In spite of observing these three criteria, the criterion “game”, which is composed of seven variables (service area, stroke effectiveness, kinds of technical and tactical strokes, players’ hitting area, shuttle landing area, shuttle flight and rally) was selected in this research (Table 1). With the aim to provide valuable information, it was decided to merge some categories of the variables “kinds of technical and tactical strokes”, “players’ hitting area” and “shuttle landing area”.

### 2.4. Procedure

Two cameras located at both court backgrounds were used, which were calibrated at a height of 2.40 m above ground and at a distance of 6.40 m from the baseline. The “Kinovea 0.8.15” computer software was chosen using a double screen and a “perspective grid” tool to delimit the court format in order to analyze the recorded matches through systematic and direct observation. The protocol recommended by Anguera et al. [46] for continuous recording of all technical and tactical behaviors was carried out, in the same way it was used in tennis by Giménez-Egido et al. [27].

### 2.5. Data Quality Control

The data was assembled by two observers who have graduated in primary education with a focus on physical education. Furthermore, both of them were specialized in racquet sports, specifically in badminton. Observer training was carried out following the training protocols designed previously in other studies [47,48]. The two observers performed the following training steps: (a) theoretical training by studying the use and terminology of the observational instrument; (b) practical training with the calculation of intra-observer reliability, recording 20% of behaviors in a match; (c) practical training with the calculation of inter-observer reliability, recording 33% of behaviors in another match, with 1 week apart; and (d) calculation of inter-observer and intra-observer reliability values, which were found to be in line with other investigations of performance analysis in other racquet sports such as tennis [27,49,50]. The calculation of inter-observer and intra-observer reliability was carried out through Cohen’s Kappa in all the variables, except for the variable “point duration”, for which the intraclass correlation coefficient was used. According to Altman [51], the following intervals were used in order to know the values of the inter-observer and inter-observer reliability: <0.20 poor, 0.21–0.40 fair, 0.41–0.60 moderate, 0.61–0.80 good and 0.81–1.00 very good. In the present study, the values of agreement by the two observers were “very good” in all variables. The statistical program used was the statistical package IBM SPSS Statistics 25.0 (IBM Corp., Armonk, NY, United States).

### 2.6. Data Notation

Data recording was completed via manual notation into an Excel spreadsheet. By this way, all technical and tactical actions were registered sequentially, being each row one stroke and each column a different variable under study. After that, an exploratory data analysis was done to perform the initial investigation, as well as to discover patterns and detect anomalies within the summary statistics [52]. Finally, the total number of technical and tactical actions (columns) performed by the young badminton players (rows) was counted for further statistical analysis (analysis of variance).

### 2.7. Statistical Analysis

The statistical analysis consisted of several phases: (a) a univariate descriptive analysis; (b) a *t*-test paired using null hypothesis significance testing; and (c) a paired-sample *t*-test by the Bayesian methodology. First, a descriptive analysis of the counts and percentages was realized, in which the mean values and standard deviation of each analyzed category were calculated. Secondly, an unconditional analysis model was conducted, using Student’s *t*-test for paired samples, establishing statistically significant differences in *p* < 0.05 [53]; this because the normality assumptions were satisfied (Kolmogorov–Smirnov test). In the third phase, the Bayesian methodology was carried out on all the variables. The Bayesian methodology (based on the quantification of the relative degree of evidence for supporting two rival hypotheses, the null hypothesis (H0) vs. alternative hypothesis (H1), by means of the Bayesian factor (BF10) [54,55]) has been recently suggested as an alternative to the traditional frequentist statistics (based on confidence intervals and *p* values) for hypothesis testing due to (among others) the following benefits: the BF10 quantifies evidence that the data provide for H0 vs. H1; the BF10 can quantify evidence in favor of H0; and the BF10 is not “violently biased” against H0 [56,57]. The BF10 was interpreted using the evidence categories suggested by Lee and Wagenmakers [58]: <1/100 = extreme evidence for H0; from 1/100 to <1/30 = very strong evidence for H0; from 1/30 to <1/10 = strong evidence for H0; from 1/10 to <1/3 = moderate evidence for H0; from 1/3 to <1 anecdotal evidence for H0; from 1 to 3 = anecdotal evidence for H1; from >3 to 10 = moderate evidence for H1; from >10 to 30 = strong evidence for H1; from >30 to 100 = very strong evidence for H1; and >100 extreme evidence for H1. The statistical analysis was performed using the spreadsheet “Jamovi 1.1.5” based on the graphical user interface R.

## 3. Results

Table 2 shows the differences between B and MB in respect of the usage percentage and the stock count technical and tactical according to service area.

The results indicate that there is a probability of 9.964 to find differences in the number of actions in the advantage zone in B compared to MB, with evidence at a Strong qualitative level. In the same way, the number of actions in the deuce zone has a probability of 6.909 to find differences in B with respect to MB, with evidence at a Moderate qualitative level.

The Table 3 presents the differences between B and MB in terms of the usage percentage and the stock count technical and tactical in accordance with stroke effectiveness.

Data show that, in the percentage, both in B and MB the most determined action is total continuity, followed by error, partial continuity and winner. The results obtained indicate that there is a probability of 14.642 to find differences in the number of actions in total continuity in B compared to MB, with evidence at a Strong qualitative level. It can also be observed a probability of 4.982 to find a high percentage of winners in B with respect to MB, with evidence at a Moderate qualitative level. There are no notable differences in the rest of the variables, which show evidence at an Anecdotal qualitative level.

Table 4 shows the differences between B and MB with regard to the usage percentage and the stock count technical and tactical as depending on the kinds of technical and tactical strokes.

The data reveal that, in the percentage, the most determined strokes in B are forehand clear, followed by forehand service and forehand smash. Thus, in general, the results show a probability of 234.335 (percentage) and 179.109 (number of actions) to find differences in total forehand in B compared to MB, with evidence at an Extreme qualitative level. In the case of total backhand, the results indicate a probability of 234.359 (percentage) and 43.227 (number of actions) to find differences in MB with respect to B, with evidence at an Extreme and Very Strong qualitative level, respectively.

In a more detailed way, it highlights the probability to find differences both in percentage and number of actions in MB compared to B in forehand drop (30,579.697–3094.865), backhand drop (7397.641–601.093) and backhand lob (142.644–111.347), in all cases, with evidence at an Extreme qualitative level. In turn, there is a probability of 35.934 to find differences in the percentage forehand drive in MB in relation to B, with evidence at a Very Strong qualitative level. Likewise, there is a probability to see differences both in the percentage and number of actions in MB with respect to B in special strokes (161.711–72.056), with evidence at an Extreme and Very Strong qualitative level, respectively. The probability to find differences both in percentage (3259.116) and number of actions (531.678) in B concerning to MB in forehand clear should also be emphasized, with evidence at an Extreme qualitative level. Finally, the probability in the percentage (8.023) and number of actions (17.292), to find differences in smash in jump in MB compared to B, should be noted, with evidence at a Moderate and Strong qualitative level, respectively.

Table 5 presents the differences between B and MB in terms of the usage percentage and the stock count technical and tactical in consideration of player hitting area.

Overall, the obtained results show a probability of 13.631 to find differences in number of hitting actions in the deuce zone in B compared to MB, with evidence at a Strong qualitative level. In relation to the court area, there is a probability to find differences in MB with respect to B in hitting percentage in the service zone (1380.579) and close to the net (35.667), with evidence at an Extreme and Strong qualitative level, respectively. On the other hand, there is a probability, both in percentage (603,088.64) and in number of actions (183,255.439), to hit the inside court in B in relation to MB, either way with evidence at an Extreme qualitative level. In more detail, there is a probability in the percentage (400.784) and in the number of actions (390.436) to hit in the baseline and sideline in MB compared to B, with evidence at an Extreme qualitative level. In turn, there is a probability to find differences in MB compared to B in the percentage of hitting in the service area, more precisely, in the advantage zone (80.777), deuce zone (11.691) and close to the net in deuce zone (10.887), with evidence at a Very Strong (service area advantage zone) and Strong qualitative level.

Table 6 shows the mean and standard deviation, as well as the *p*-value of the usage percentage and the stock count technical and tactical in B and MB according to shuttle landing area.

In general, the results in Table 6 indicate that there is a probability of finding differences in errors in the percentage (264.660) and number of actions (3.094) that are not in the net in MB compared to B, with evidence at an Extreme and Moderate qualitative level, respectively. In such a way, there is a probability in the percentage (264.660) and number of actions (236.083) to find differences in net errors in B with respect to MB, with evidence at an Extreme qualitative level. The probability in the percentage (98.766) and number of actions (45.035) in shuttle landing area (inside court) in B with regard to MB should also be emphasized, with evidence at a Very Strong qualitative level.

Table 7 presents the mean and standard deviation, as well as the *p*-value of the usage percentage and the stock count technical and tactical in B and MB in accordance with shuttle flight.

The results in Table 7 bring to light that, in percentage, both in B and MB, the most determined shuttle flight is the crossed one, followed by the straight one and others. It should also be taken into consideration that there is a probability of 14.364 to find differences in the number of actions that are crossed flights in B regarding to MB, with evidence at a Strong qualitative level. In addition, there is a probability of 9.389 to find differences in the percentage in other flights in MB concerning B, with evidence at a Strong qualitative level.

Table 8 shows the mean and standard deviation, as well as the *p*-value, of the usage percentage and the stock count technical and tactical in B and MB according to rally.

Table 8 results reveal that, in percentage, both in B and MB, the most frequent rally range is 2–5, followed by 6–9, 1 and, lastly, +9. It should be noted that there is evidence at an Anecdotal qualitative level of different rally duration options, both from the point of view of the percentage and the number of actions.

## 4. Discussion

The main purpose of the present research was to examine the incidence of MB by reducing the court dimensions (from 13.40 m × 5.18 m to 11.8 m × 5.18 m) and net height (from 1.55 m to 1.30 m), and observing the differences with the current under-11 badminton players’ competition (B) in the following technical and tactical variables: (a) service area; (b) stroke effectiveness; (c) kinds of technical strokes; (d) players’ hitting area; (e) shuttle landing area; (f) shuttle flight; and (g) rally length. Considering other similar studies on junior tennis [25,27,59], scaling task constraints (reducing net height and court size) in a real-game context for under-11 badminton players seems to promote optimal learning opportunities. Hence, MB encourage players to seek new offensive play patterns by hitting different kind of strokes that enhance their behavioral variability, even if their effectiveness does not improve. Furthermore, according to the ideas and concepts developed by Torrents et al. [60], MB could help the acquisition of motor creativity, by affording a degree of freedom related to the increase of hitting variability and reducing conscious awareness in a real-game context using proper task constraints. Overall, MB appears to be beneficial for optimal children’s sport and personal development according to NLP principles and “scaling junior sport”.

Promoting desirable motor skills is an important aspect in “scaling junior sport” [12], so examining whether there are imbalances in play patterns is a key aspect in junior sport. Hence, the number of different kinds of strokes was compared, and in both competitions more forehand strokes were hit. However, a greater use of backhands can be observed in MB. These results coincide with the evidence found in other studies, which revealed the proliferation of the backhand stroke when reducing court size [21,49]. The emergence of special strokes was also analyzed, noting that it is approximately 167 times more likely to occur in MB than in B, although there was not much difference in the total percentage between B (0.36%) and MB (1.37%). These results coincide with those obtained in tennis [27] or dance [61], which showed that creativity processes are related at a theoretical level to motor variability in problem solving [60].

On the other hand, previous studies [23,24,27,62] on this topic and NLP [2] indicated that it is important to design children’s competitions that facilitate an offensive style of playing [23,24,27,62]. Following this line, the distance reduction between the baseline and the net and the reduction in the net height can explain the increase of strokes executed in the service zone and close to the net (more offensive areas) in MB. Moreover, the players used more hits classified as offensive (forehand and backhand drop and smash in jump) by the Spanish Badminton Federation [37]. Otherwise, the likelihood of performing forehand clears is higher in B than in MB (BF10 = 3259.11); this result showed that the players adopted a defensive playing style, because it is a very common defensive stroke. Consequently, players use it to avoid taking risks during the game.

Analyzing rally length, similar values were observed between B and MB, in addition to the fact that the players were hitting mainly cross strokes. Two possible explanations for these results may be as follows: the players’ lack of experience to perceive relevant information during match-play to change their play patterns; or excessive use of forehand clear in B, not promoting motor variability. Giménez Egido [27] found similar results in tennis, while the study conducted by Nor Azmil et al. [40] regarding badminton found that players increased their rally length by reducing the net height, court size and racket size. These results from two studies may have different meanings: Fitzpatrick et al. [49] indicated that an improved rally length leads to increased learning; nevertheless, Schimodffer et al. [62] showed evidence that a longer duration of the rally implies a more defensive playing style. According to NLP [2], the most desirable is to foster an offensive play style that enhances learning processes at the formative stage. In this line, the category total continuity obtained a higher probability of success in B than in MB. The results confirm our hypotheses, except for the increase in stroke effectiveness. A possible explanation could be that the training tasks are still based on linear models, outside the pedagogical principles of NLP, although the players usually play under MB conditions.

Finally, several studies highlighted that a strong limiting factor for learning is net height in child tennis [21,23,24,26,27] and badminton [40] players. These studies indicate that it is necessary to decrease the net height according to the players’ need and characteristics for optimal development, as a greater number of errors occur when players hit inside the net. In accordance with these studies, the results showed that the percentage of errors made in the net are higher in B due to the net height.

Although this study shows strong evidence, the current study had some limitations: the cross-sectional design provided only the short-term effect of these modified competitions; and the sample was composed of only elite junior players. In the future, it would be interesting to reproduce this study with different players’ characteristic.

According to Giménez-Egido et al. [23] and Buszard et al. [11], future research in junior badminton should focus on designing nonlinear competition formats, without relying exclusively on the physical maturity or age of the players; for example, using oversize racquets with slower shuttles in MB conditions. In addition, this type of study should be applied by assessing the psychological factors, such as self-efficacy or satisfaction, that mediate the adequate acquisition of motor and behavioral skills.

## 5. Conclusions

Changes in methodological approaches, just as the implementation of scaling constraints in junior sports, enhance the acquisition of desirable motor and behavioral skills. The findings of this study indicated that MB facilitate the use of different kind of strokes that promote motor variability by reducing conscious control when performed in a real-game context. Thus, MB improves the players’ capacity to explore new play patterns, and such problem-solving may induce creativity behaviors. Therefore, MB affords optimal learning opportunities according to a contemporaneous pedagogical approach. Taking into account the aforementioned, it can be said that the application of modified equipment and playing spaces in a competitive context (in this case, reducing the court dimensions as well as the net height) could have benefits for the learning processes of junior players.

In terms of practical application, this type of research can provide valuable information in order to use the most appropriate kind of methodology in training, as well as facing competition in a more productive way.

## Figures and Tables

**Table 1:** Macro variables, micro variables, initial category of the observational instrument (initial category) and their final transformation for this study (final category).

Macro Variable: Service Area	
**Initial category**	**Final category**
Advantage service area	Advantage zone
Deuce service area	Deuce zone
**Macro Variable: Stroke Effectiveness**	
**Initial category**	**Final category**
Winner	Winner
Total continuity	Total continuity
Partial continuity	Partial continuity
Error	Error
**Macro Variable: Kinds of Technical and Tactical Strokes**	
**Micro Variable: Basic Strokes**	
**Initial category**	**Final category**
Right serve	Forehand serve
Reverse serve	Backhand serve
Clear from right to high hand	Forehand clear
Clear from right to medium height	
Clear from right to low hand	
Clear from left to high hand	Backhand clear
Clear from left to medium height	
Clear from left to low hand	
Right drop	Forehand drop
Left drop	Backhand drop
Right smash	Forehand smash
Left smash	Backhand smash
Smash in jump	Smash in jump
Drive from right to high hand	Forehand drive
Drive from right to medium height	
Drive from left to high hand	Backhand drive
Drive from left to medium height	
Net drop from right to medium height	Forehand net drop
Net drop from right to low hand	
Net drop from left to medium height	Backhand net drop
Net drop from left to low hand	
Lob from right to medium height	Forehand lob
Lob from right to low hand	
Lob from left to medium height	Backhand lob
Lob from left to low hand	
Micro variable special strokes	
Right brush	Total special strokes
Left brush	
Right kill	
Left kill	
Right push	
Left push	
**Macro Variable: Players’ Hitting Area**	
**Initial category**	**Final category**
Baseline, out of court	Baseline and sideline
Deuce sideline	
Advantage sideline	
Inside the court, serve and background zone, in the left area	Inside court, service area and baseline, advantage zone
Inside the court, serve and background zone, in the central area	
Inside the court, serve and background zone, in the central area	Inside court, service area and baseline, deuce zone
Inside the court, serve and background zone, in the right area	
Serve zone, in the left area	Service area, advantage zone
Serve zone, in the central area	
Serve zone, in the central area	Service area, deuce zone
Serve zone, in the right area	
Near the net, in the left area	Close the net, advantage zone
Near the net, in the central area	
Near the net, in the central area	Close the net, deuce zone
Near the net, in the right area	
**Macro Variable: Shuttle Landing Area**	
**Initial category**	**Final category**
Net error	Net error
Background error	Depth error
Deuce sideline error	Sideline error
Advantage sideline error	
Inside the court, serve and background zone, in the left area	Inside court, service area and baseline, advantage zone
Inside the court, serve and background zone, in the central area	
Inside the court, serve and background zone, in the central area	Inside court, service area and baseline, deuce zone
Inside the court, serve and background zone, in the right area	
Serve zone, in the left area	Service area, advantage zone
Serve zone, in the central area	
Serve zone, in the central area	Service area, deuce zone
Serve zone, in the right area	
Near the net, in the left area	Close the net, advantage zone
Near the net, in the central area	
Near the net, in the central area	Close the net, deuce zone
Near the net, in the right area	
Own field error	Own field and roof
Roof error	
**Macro Variable: Shuttle Flight**	
**Initial category**	**Final category**
Parallel	Straight
Cross	Crossed
Other	Other
**Macro Variable: Rally Length**	
**Initial category**	**Final category**
1 Stroke	1
2 to 5	2–5
6 to 9	6–9
Over 9	+9

**Table 2:** Bayesian analysis of usage percentage and stock count technical and tactical according to service area.

Percentage							Number of Actions					
	Badminton	Mini-Badminton	*p-*Value	Error %	Bayesian Factor	δ	Badminton	Mini-Badminton	*p-*Value	Error %	Bayesian Factor	δ
Advantage zone	50.78 ± 4.60	51.71 ± 5.68	0.464	0.01216	BF01 = 3.873	−0.128	30.12 ± 12.38	23.68 ± 6.14	0.004	0.00112	BF10 = 9.964	0.542
Deuce zone	49.22 ± 4.60	48.29 ± 5.58	0.464	0.01216	BF01 = 3.873	−0.125	29.43 ± 12.46	22.14 ± 6.11	0.006	7.37 × 10^−6^	BF10 = 6.909	0.512

**Table 3:** Bayesian analysis of usage percentage and stock count technical and tactical in accordance with stroke effectiveness.

Percentage							Number of Actions					
	Badminton	Mini-Badminton	*p-*Value	Error %	Bayesian Factor	δ	Badminton	Mini-Badminton	*p-*Value	Error %	Bayesian Factor	δ
Winner	9.82 ± 4.74	9.77 ± 3.75	0.960	0.00407	BF01 = 4.982	0.010	5.49 ± 2.88	4.49 ± 2.07	0.074	1.28 × 10^−5^	BF01 = 1.107	0.318
Total continuity	66.15 ± 5.42	63.26 ± 6.54	0.037	1.09 × 10^−5^	BF10 = 1.552	0.376	39.89 ± 17.33	29.00 ± 7.95	0.003	5.12 × 10^−4^	BF10 = 14.642	0.577
Partial continuity	10.71 ± 3.23	12.39 ± 3.47	0.062	1.23 × 10^−5^	BF01 = 1.039	−0.335	6.58 ± 3.55	5.69 ± 2.05	0.179	0.02528	BF01 = 2.132	0.232
Error	13.30 ± 4.87	14.56± 5.18	0.205	0.02423	BF01 = 2.341	−0.222	7.58 ± 3.77	6.64 ± 2.62	0.128	1.45 × 10^−5^	BF01 = 1.671	0.270

**Table 4:** Bayesian analysis of usage percentage and stock count technical and tactical according to kinds of technical and tactical strokes.

Percentage							Number of Actions					
	Badminton	Mini-Badminton	*p-*Value	Error %	Bayesian Factor	δ	Badminton	Mini-Badminton	*p-*Value	Error %	Bayesian Factor	δ
Forehand service	18.34 ± 7.75	18.75 ± 9.52	0.759	0.00539	BF01 = 4.771	−0.054	11.25 ± 6.30	8.71 ± 4.78	0.010	8.21 × 10^−6^	BF10 = 4.481	0.474
Backhand service	4.61 ± 8.94	5.64 ± 9.31	0.340	0.01744	BF01 = 3.250	−0.170	2.13 ± 4.00	2.43 ± 4.05	0.531	0.00995	BF01 = 4.145	−0.106
Forehand clear	38.02 ± 9.26	27.74 ± 11.31	0.000	1.29 × 10^−6^	BF10 = 3259.116	0.989	22.34 ± 10.19	12.33 ± 4.79	0.000	7.11 × 10^−6^	BF10 = 531.678	0.856
Backhand clear	3.27± 2.25	4.37 ± 2.97	0.113	1.41 × 10^−5^	BF01 = 1.522	−0.275	1.94 ± 1.67	2.05 ± 1.64	0.804	0.00491	BF01 = 4.845	−0.041
Forehand drop	1.03 ± 2.46	6.20 ± 4.30	0.000	2.59 × 10^−7^	BF10 = 30,579.697	−1.161	0.48 ± 0.96	2.99 ± 2.56	0.000	1.40 × 10^−6^	BF10 = 3094.865	−0.992
Backhand drop	0.08 ± 0.33	2.13 ± 1.83	0.000	4.11 × 10^−7^	BF10 = 7397.641	−1.051	0.06 ± 0.26	1.05 ± 1.06	0.000	6.58 × 10^−6^	BF10 = 601.093	−0.861
Forehand smash	16.05 ± 5.50	4.46 ± 2.94	0.000	3.04 × 10^−12^	BF10 = 1.256 × 10^8^	−1.868	9.92 ± 6.09	2.00 ± 1.40	0.000	2.12 × 10^−7^	BF10 = 54,543.142	1.201
Backhand smash	0.20 ± 0.38	0.12 ± 0.36	0.376	0.01578	BF01 = 3.446	0.152	0.14 ± 0.26	0.05 ± 0.15	0.073	1.28 × 10^−5^	BF01 = 1.094	0.322
Smash in jump	2.33 ± 3.73	5.32 ± 5.58	0.005	7.09 × 10^−6^	BF10 = 8.023	−0.522	1.09 ± 1.32	2.49 ± 2.69	0.002	3.52 × 10^−4^	BF10 = 17.292	−0.584
Forehand drive	7.93 ± 3.88	13.25 ± 5.73	0.001	4.76 × 10^−5^	BF10 = 35.934	−0.650	4.90± 3.02	6.08 ± 3.02	0.182	0.02518	BF01 = 2.154	−0.235
Backhand drive	0.38 ± 0.60	0.82 ± 0.99	0.040	1.11 × 10^−5^	BF10 = 1.482	−0.372	0.21 ± 0.29	0.39 ± 0.46	0.074	1.29 × 10^−5^	BF01 = 1.111	−0.314
Forehand net drop	2.88 ± 2.56	2.48 ± 3.21	0.440	0.01304	BF01 = 3.768	0.133	2.01 ± 2.00	1.21 ± 1.51	0.003	5.94 × 10^−4^	BF10 = 13.659	0.573
Backhand net drop	0.00 ± 0.00	1.23 ± 1.43	0.000	--	--	--	0.00 ± 0.00	0.59 ± 0.68	0.000	--	--	--
Forehand lob	4.48 ± 3.19	3.82 ± 3.59	0.382	0.01550	BF01 = 3.479	0.151	2.86 ± 2.10	1.76 ± 1.57	0.004	0.00115	BF10 = 9.856	0.541
Backhand lob	0.02 ± 0.13	2.30 ± 2.79	0.000	1.03 × 10^−5^	BF10 = 142.644	−0.759	0.01± 0.06	1.06 ± 1.31	0.000	8.83 × 10^−6^	BF10 = 111.347	−0.735
Special strokes	0.36 ± 0.55	1.37 ± 1.27	0.000	1.07 × 10^−5^	BF10 = 161.711	−0.762	0.20 ± 0.30	0.62 ± 0.60	0.000	4.00 × 10^−6^	BF10 = 72.056	−0.702
Total forehand	91.00 ± 10.22	82.34 ± 13.78	0.000	1.04 × 10^−5^	BF10 = 234.335	0.762	53.94 ± 22.89	35.70 ± 11.06	0.000	1.07 × 10^−5^	BF10 = 179.109	0.772
Total Backhand	9.00 ± 10.22	17.66 ± 13.78	0.000	1.04 × 10^−5^	BF10 = 234.359	−0.790	4.52 ± 4.54	7.63 ± 5.92	0.001	2.46 × 10^−5^	BF10 = 43.227	−0.663

**Table 5:** Bayesian analysis of usage percentage and stock count technical and tactical in consideration of players’ hitting area.

Percentage							Number of Actions					
	Badminton	Mini-Badminton	*p-*Value	Error %	Bayesian Factor	δ	Badminton	Mini-Badminton	*p-*Value	Error %	Bayesian Factor	δ
Baseline and sideline	0.01 ± 0.08	1.28± 1.42	0.000	8.36 × 10^−6^	BF10 = 400.784	−0.833	0.01 ± 0.06	0.58 ± 0.64	0.000	8.48 × 10^−6^	BF10 = 390.436	−0.823
Inside court service area and baseline advantage zone	18.15 ± 4.23	10.89 ± 3.89	0.000	2.42 × 10^−7^	BF10 = 38,323.012	1.176	10.34 ± 3.95	4.84 ± 1.81	0.000	1.10 × 10^−8^	BF10 = 105,917.567	1.259
Inside court service area and baseline deuce zone	15.37 ± 5.21	7.94 ± 4.11	0.000	1.21 × 10^−8^	BF10 = 99,421.405	1.150	8.69 ± 3.84	3.62 ± 2.04	0.000	2.42 × 10^−7^	BF10 = 37,942.293	1.185
Service area advantage zone	32.65 ± 5.64	38.13 ± 6.29	0.000	5.26 × 10^−6^	BF10 = 80.777	−0.715	19.55 ± 9.27	17.44 ± 5.18	0.254	0.02185	BF01 = 2.700	0.196
Service area deuce zone	27.34 ± 4.87	30.96 ± 5.93	0.003	8.20 × 10^−4^	BF10 = 11.691	−0.562	16.48 ± 7.64	14.19± 4.56	0.132	1.46 × 10^−5^	BF01 = 1.714	0.266
Close to the net advantage zone	3.64 ± 3.12	5.95 ± 4.56	0.014	8.72 × 10^−6^	BF10 = 3.511	−0.455	2.55 ± 2.32	2.77 ± 2.21	0.599	0.00815	BF01 = 4.379	−0.087
Close to the net deuce zone	2.82 ± 2.23	4.84 ± 3.61	0.004	9.46 × 10^−4^	BF10 = 10.887	−0.547	1.94 ± 1.76	2.37 ± 1.84	0.174	0.02545	BF01 = 2.089	−0.240
Inside court	33.53 ± 8.20	19.09 ± 6.03	0.000	3.87 × 10−10	BF10 = 603,088.64	1.408	19.04 ± 7.17	8.46 ± 3.14	0.000	4.44 × 10^−9^	BF10 = 183,255.439	1.297
Service square	60.00 ± 7.76	69.98 ± 7.57	0.000	3.66 × 10^−6^	BF10 = 1380.579	−0.925	36.02 ± 16.26	31.63 ± 8.78	0.178	0.02532	BF01 = 2.121	0.235
Close to the net	6.47 ± 4.54	10.93 ± 7.35	0.001	4.88 × 10^−5^	BF10 = 35.667	−0.647	4.49 ± 3.69	5.14 ± 3.69	0.261	0.02148	BF01 = 2.750	−0.193
Advantage zone	54.46 ± 5.71	55.67 ± 5.97	0.341	0.0174	BF01 = 3.255	−0.165	32.44 ± 13.84	25.06 ± 6.26	0.006	7.18 × 10^−6^	BF10 = 7.651	0.521
Deuce zone	45.54 ± 5.71	44.33 ± 5.97	0.341	0.0174	BF01 = 3.255	−0.167	27.11 ± 11.57	20.18 ± 6.07	0.003	5.97 × 10^−4^	BF10 = 13.631	0.573

**Table 6:** Bayesian analysis of usage percentage and stock count technical and tactical according to shuttle landing area.

Percentage							Number of Actions					
	Badminton	Mini-Badminton	*p-*Value	Error %	Bayesian Factor	δ	Badminton	Mini-Badminton	*p-*Value	Error %	Bayesian Factor	δ
No net error	51.65 ± 14.44	67.93 ± 12.48	0.000	1.70 × 10^−5^	BF10 = 264.660	−0.824	3.97 ± 2.32	4.43 ± 1.89	0.322	0.0268	BF01 = 3.094	−0.169
Net error	48.35 ± 14.44	32.06 ± 12.48	0.000	1.70 × 10^−5^	BF10 = 264.660	0.820	3.59 ± 1.93	2.15 ± 1.16	0.000	1.84 × 10^−5^	BF10 = 236.083	0.808
Net error	48.35 ± 14.44	32.06 ± 12.48	0.000	1.70 × 10^−5^	BF10 = 264.660	0.820	3.59 ± 1.93	2.15 ± 1.16	0.000	1.84 × 10^−5^	BF10 = 236.083	0.808
Depth error	9.99 ± 10.26	15.32 ± 11.39	0.063	2.03 × 10^−5^	BF10 = 1.038	−0.337	0.69 ± 0.67	1.16 ± 0.99	0.011	1.44 × 10^−5^	BF10 = 4.362	−0.484
Sideline error	17.87 ± 12.73	22.61 ± 13.49	0.137	2.36 × 10^−5^	BF01 = 1.731	−0.261	1.22 ± 0.87	1.52 ± 1.20	0.264	0.0289	BF01 = 2.725	−0.202
Own field and roof	23.79 ± 14.35	30.00 ± 16.62	0.127	2.33 × 10^−5^	BF01 = 1.639	−0.270	2.06 ± 1.85	1.75 ± 0.76	0.334	0.0263	BF01 = 3.165	0.170
Inside court service area and baseline advantage zone	20.63 ± 12.86	16.10 ± 11.39	0.118	2.29 × 10^−5^	BF01 = 1.551	0.282	1.21 ± 0.88	0.72 ± 0.60	0.019	1.60 × 10^−5^	BF10 = 2.767	0.441
Inside court service area and baseline deuce zone	28.44 ± 14.99	20.43 ± 14.04	0.018	1.60 × 10^−5^	BF10 = 2.822	0.440	1.78 ± 1.17	0.96 ± 0.69	0.001	1.83 × 10^−5^	BF10 = 42.002	0.676
Service area advantage zone	11.56 ± 11.94	16.28 ± 9.43	0.129	2.33 × 10^−5^	BF01 = 1.658	−0.270	0.60 ± 0.46	0.68 ± 0.42	0.580	0.0181	BF01 = 4.251	−0.099
Service area deuce zone	16.91 ± 12.05	20.25 ± 14.10	0.326	0.0266	BF01 = 3.119	−0.174	1.02 ± 0.86	0.99 ± 0.90	0.872	0.0136	BF01 = 4.848	0.027
Close to the net advantage zone	9.29 ± 9.55	10.39 ± 13.14	0.731	0.0151	BF01 = 4.642	−0.059	0.47 ± 0.41	0.41 ± 0.46	0.655	0.0164	BF01 = 4.468	0.077
Close to the net deuce zone	13.16 ± 12.55	16.54 ± 15.57	0.301	0.0276	BF01 = 2.967	−0.180	0.76 ± 0.73	0.69 ± 0.65	0.456	0.0218	BF01 = 3.781	0.133
Inside court	49.07 ± 13.74	36.54 ± 14.01	0.000	2.84 × 10^−5^	BF10 = 98.766	0.746	2.99 ± 1.69	1.68 ± 0.86	0.001	2.07 × 10^−5^	BF10 = 45.035	0.678
Service area	28.48 ± 11.47	36.53 ± 14.60	0.019	1.61 × 10^−5^	BF10 = 2.679	−0.437	1.63 ± 0.97	1.67 ± 1.06	0.895	0.0134	BF01 = 4.867	−0.025
Close to the net	22.45 ± 14.37	26.93 ± 16.72	0.306	0.0274	BF01 = 2.996	−0.186	1.23 ± 0.80	1.10 ± 0.80	0.421	0.0231	BF01 = 3.620	0.141
Advantage zone	41.49 ± 22.84	42.77 ± 17.84	0.759	0.0147	BF01 = 4.694	−0.053	2.28 ± 1.24	1.80 ± 0.89	0.099	2.22 × 10^−5^	BF01 = 1.363	0.299
Deuce zone	58.51 ± 22.84	57.22 ± 17.84	0.759	0.0147	BF01 = 4.694	−0.056	3.57 ± 2.16	2.64 ± 1.42	0.020	1.63 × 10^−5^	BF01 = 2.594	0.429

**Table 7:** Bayesian analysis of usage percentage and stock count technical and tactical in accordance with shuttle flight.

Percentage							Number of Actions					
	Badminton	Mini-Badminton	*p-*Value	Error %	Bayesian Factor	δ	Badminton	Mini-Badminton	*p-*Value	Error %	Bayesian Factor	δ
Straight	40.84 ± 6.00	40.02 ± 5.26	0.588	0.00842	BF01 = 4.343	0.092	24.54 ± 11.12	18.50 ± 5.65	0.007	7.59 × 10^−6^	BF10 = 6.148	0.508
Crossed	56.62 ± 6.85	56.01 ± 5.05	0.644	0.00718	BF01 = 4.511	0.079	33.50 ± 13.53	25.61 ± 6.55	0.003	5.33 × 10^−4^	BF10 = 14.364	0.573
Other	2.55 ± 2.47	3.97 ± 2.08	0.004	0.00126	BF10 = 9.389	−0.546	1.62 ± 1.55	1.71 ± 0.77	0.704	0.00613	BF01 = 4.661	−0.065

**Table 8:** Bayesian analysis of usage percentage and stock count technical and tactical according to rally.

Percentage							Number of Actions					
	Badminton	Mini-Badminton	*p-*Value	Error %	Bayesian Factor	δ	Badminton	Mini-Badminton	*p-*Value	Error %	Bayesian Factor	δ
1	10.58 ± 5.89	10.66 ± 7.01	0.954	0.00408	BF01 = 4.980	−0.009	1.36 ± 0.80	1.29 ± 0.92	0.570	0.00888	BF01 = 4.283	0.100
2–5	61.12 ± 10.05	65.26 ± 8.17	0.041	1.12 × 10^−5^	BF10 = 1.431	−0.371	8.02 ± 3.26	7.21 ± 2.33	0.139	1.48 × 10^−5^	BF01 = 1.779	0.256
6–9	23.11 ± 9.73	20.50 ± 7.55	0.321	0.01841	BF01 = 3.135	−0.172	3.26 ± 1.87	2.29 ± 1.17	0.027	1.01 × 10^−5^	BF10 = 2.009	0.408
+9	5.18 ± 5.27	3.58 ± 3.48	0.159	1.52 × 10^−5^	BF01 = 1.960	0.245	0.65 ± 0.65	0.38 ± 0.40	0.082	1.31 × 10^−5^	BF01 = 1.196	0.312
